# The Prevalence of Diabetes and Prediabetes in the Adult Population of Jeddah, Saudi Arabia- A Community-Based Survey

**DOI:** 10.1371/journal.pone.0152559

**Published:** 2016-04-01

**Authors:** Suhad M. Bahijri, Hanan A. Jambi, Rajaa M. Al Raddadi, Gordon Ferns, Jaakko Tuomilehto

**Affiliations:** 1 Department of Clinical Biochemistry - Faculty of Medicine- King Abdulaziz University, Jeddah, Saudi Arabia; 2 Saudi Diabetes Study Research Group- King Fahd Medical Research Center - King Abdulaziz University, Jeddah, Saudi Arabia; 3 Department of Food and Nutrition- Home Economics, King Abdulaziz University, Jeddah, Saudi Arabia; 4 Ministry of Health, Public Health Directorate, Jeddah, Saudi Arabia; 5 Division of Medical Education, Brighton and Sussex Medical School, Mayfield House, Falmer, Brighton, United Kingdom; 6 Center for Vascular Prevention, Danube University Krems, Krems, Austria; 7 Dasman Diabetes Institute, Dasman, Kuwait; University of Tolima, COLOMBIA

## Abstract

**Background:**

Type 2 (T2DM) is believed to be common in Saudi Arabia, but data are limited. In this population survey, we determined the prevalence of T2DM and prediabetes.

**Materials and Methods:**

A representative sample among residents aged ≥ 18 years of the city of Jeddah was obtained comprising both Saudi and non-Saudi families (N = 1420). Data on dietary, clinical and socio-demographic characteristics were collected and anthropometric measurements taken. Fasting plasma glucose and glycated hemoglobin (HbA1c) were used to diagnose diabetes and prediabetes employing American Diabetes Association criteria. Multiple logistic regression analysis was used to identify factors associated with T2DM.

**Results:**

Age and sex standardized prevalence of prediabetes was 9.0% (95% CI 7.5–10.5); 9.4% (7.1–11.8) in men and 8.6% (6.6–10.6) in women. For DM it was 12.1% (10.7–13.5); 12.9% (10.7–13.5) in men and 11.4% (9.5–13.3) in women. The prevalence based on World Population as standard was 18.3% for DM and 11.9% for prediabetes. The prevalence of DM and prediabetes increased with age. Of people aged ≥50 years 46% of men and 44% of women had DM. Prediabetes and DM were associated with various measures of adiposity. DM was also associated with and family history of dyslipidemia in women, cardiovascular disease in men, and with hypertension, dyslipidemia and family history of diabetes in both sexes.

**Discussion:**

Age was the strongest predictor of DM and prediabetes followed by obesity. Of people aged 50 years or over almost half had DM and another 10–15% had prediabetes leaving only a small proportion of people in this age group with normoglycemia. Since we did not use an oral glucose tolerance test the true prevalence of DM and prediabetes is thus likely to be even higher than reported here. These results demonstrate the urgent need to develop primary prevention strategies for type 2 diabetes in Saudi Arabia.

## Introduction

The prevalence of diabetes mellitus (DM) is reported to be rising globally in parallel with an increasing prevalence of obesity [1]. Saudi Arabia and other Middle Eastern countries have a particularly high prevalence of both conditions [1–4]. DM is irreversible once established. It is a slowly developing but progressive condition; and it can take many years to progress from prediabetic to diabetic state without interventions [5]. Therefore, attempting to prevent this progression or at least to delay it should be a superior national health strategy than only attempting to manage the disease after it is established [6]. An established basis for a prevention strategy is to identify common risk modifiable factors that have the greatest contribution to morbidity, and develop community based programmes for their prevention and control. This has been discussed thoroughly in the " European Evidence-Based Guideline for the Prevention of Type 2 Diabetes" [7], with steps and strategies needed to implement prevention outlined elegantly by Lindstrom et al. [8]. Although it is obvious that it is important to know the true magnitude of the major disease such as DM, reliable data on its prevalence are lacking for most countries. Previous studies in Saudi Arabia have been not been based on representative population samples, and have used fasting glucose values only.

For type 2 DM (T2DM) the diagnosis is based on elevated glucose concentration in blood circulation. Therefore, it is necessary to carry out an assessment with glucose determination in the target population in order to determine the prevalence of T2DM and other disorders of glucose metabolism. With this in mind, the main objective of our study was to determine the prevalence of T2DM and prediabetes in a representative population sample among residents of the city of Jeddah using the methodology recommended by American Diabetes Association (ADA). In addition, we also aimed to identify factors associated with variation in these conditions in order to formulate hypotheses of the major risk factors of T2DM in this population and develop appropriate preventive intervention strategies.

## Materials and Methods

Using earlier published prevalence data [2] the estimated sample size required for this study was calculated to be 1350. The study was approved by the Committee on the Ethics of Human Research at the ‘‘Faculty of Medicine- King Abdulaziz University”.

### Sampling Methodology

The World Health Organization (EPI) cluster survey design has become the method of preference in the field to measure vaccination coverage and other indicators such as prevalence studies of diseases [9]. Therefore, mapping cluster sampling was adopted in sampling of the current study employing the digital georeferenced map for Jeddah Governorate and using GIS and ArcGis techniques. In order to get representative sample from the city, and based on the common knowledge that there are distinct socio-demographic characteristics of the population within different districts according to its location in the north or south of the city, the sample was divided equally on these subgroups.

A 3-stage stratified cluster sampling technique was adopted for the sample selection. At the first stage, Jeddah city map was divided into big clusters with radius of half a kilometer each and 36 big clusters were selected randomly (18 clusters in the north and 18 clusters in the south of Jeddah covering both organized and slum areas). At the second stage, two small clusters; with a radius of 50 meters; were chosen randomly from the list of small clusters within the big cluster, with the centroid of the small cluster being considered as the landmark for choice of the selected house. A standardized procedure was adopted to choose alternative location if the selected one was not a residential building housing families. At the third stage families were selected from each location for inclusion into the study based on type of housing. Standardized procedure was used to select families living in apartment buildings, while all residents in single occupancy residence were included in the sample. Only data obtained from people ≥ 18 years of age are included here.

### Collection of the data

An initial visit to the selected households was conducted to obtain signed informed consent and to plan the appointment for sample collection. A questionnaire was designed to collect information covering demographic (age, sex, ethnic origin, educational level, type of job if any, and family income), dietary, and lifestyle variables, as well as medical history including subjective symptoms, DM, dyslipidemia and family history of DM, dyslipidemia and/or cardiovascular disease (CVD). On the appointed visit, the participants were individually interviewed in order to complete the questionnaire, and anthropometric measurements were then taken. Height was measured bare footed to the nearest 0.5 cm using a stationary stadiometer. Weight was measured to the nearest 0.5kg while wearing light street clothing using a portable calibrated scale; these measurements were used to calculate body mass index (BMI). Waist circumference (WC) was measured at the level of the umbilicus, and hip circumference (HC) at the maximal protrusion of the gluteal muscles, both to the nearest 0.5 cm. Using WC measurements to indicate abdominal obesity the first cut-off value for increased risk was defined as >94cm for men, >80 cm for women, and the second cut-off value as >102cm for men, >88 cm for women [10–13]. The cut off value for higher risk while using the WC:HC ratio was defined as >0.95 for men, > 0.80 for women [14], and > 0.50 for both sexes while using the WC: height ratio [15–17]. Blood pressure (BP) was measured following the recommendations of the Joint National Committee, using a standard mercury sphygmomanometer with the cuff on the right upper arm [18]. Two blood pressure readings were taken; one minute apart, while the person was initially seated for 10 minutes, and the mean of the two readings was calculated. A third measurement was taken and the mean of the two closest values was calculated if there was a difference > 5 mmHg between the two first measures. Hypertension was defined as systolic BP ≥ 140 mmHg and/or diastolic BP > 90 mmHg, or current use of antihypertensive medication [18]. Random capillary plasma glucose (RPG) was measured on site using calibrated glucometers (ACCU- CHEK^®^- Model GC- Roche), and the time of last meal was recorded.

### Diagnosing diabetes and impaired glucose tolerance in suspected cases

People reporting fasting status (last meal before 8–10 hours), and having RPG of > 100mg/dl, and those less than 8 hours fasting having RPG of ≥ 126 mg/dl were given an appointment for further testing, and requested to fast for ≥ 8 hours before presenting at the laboratory. Fasting plasma glucose (FPG) and glycated hemoglobin (HbA1c) measured by calibrated glucometer (ACCU- CHEK^®^- Model GC- Roche), and calibrated Bio- Rad in2it^™^ analyzer respectively; were used to diagnose diabetes and prediabetes. People with FPG of ≥ 126 mg/dl, and/or HbA1c ≥ 6.5% were classified as diabetic. The presence of classical symptoms of diabetes confirmed diagnosis. People with FPG of 100–125 mg/dl, and /or HbA1c 5.7–6.4% were classified as having prediabetes [19]. In addition, all participants reporting previous diagnosis of DM and taking drug treatment for DM were classified as diabetic, while those with previous diagnosis but not being on glucose lowering drug treatment and having RPG < 126 mg/dl were given appointment to confirm their glycaemic status as described above.

### Statistical analysis

Analyses were performed using the SPSS statistical package version 21. Chi-square test was used to identify the association between prediabetes or DM and the independent variables. Multiple logistic regression analyses were used to adjust for confounding factors. Unadjusted and age adjusted Odds Ratio (OR) for both sexes with its 95% Confidence Interval (CI) for the predictors of DM and prediabetes were presented. Educational attainment, ethnicity, smoking, physical activity, BMI, WC, WC/HC, WC/height, percentage of body fat, diagnosis of hypertension, dyslipidemia, CVD, and family history of DM, dyslipidemia and CVD were included in the model as independent variables. Statistical significance was assigned at p < 0.05.

## Results

A total of 476 families were approached, and 390 agreed to participate indicating a response rate of 81.9%. However, not all family members agreed or were able to complete all survey measurements. Age of participants was obtained from their official documents. Mean age of the participants (N = 1420) was 36 years (SD15.4 years). Demographic and lifestyle characteristics of the population studied are presented in Table 1.

**Table 1:** Demographic and lifestyle characteristics of the participants.

	Men (% of total males)	Women (% of total females)	Total (% of total adults)
**Age (years)**:			
18–<20	165 (24.7%)	126 (16.8%)	291 (20.5%)
20–<30	137 (20.5%)	183 (24.3%)	320 (22.6%)
30–<40	97 (14.5%)	153 (20.3%)	250 (17.6%)
40–<50	101 (15.1%)	146 (19.4%)	247 (17.4%)
50–<60	105 (15.7%)	91 (12.1%)	196 (13.8%)
≥ 60	62 (9.3%)	53 (7.0%)	115 (8.1)
All adults	667 (100%)	752 (100%)	1419 (100%)
**Educational** attainment:			
≤ Primary school	37 (5.7%)	120 (16.3%)	157 (11.4%)
Intermediate school	79 (12.2%)	74 (10.1%)	153 (11.1%)
Secondary school and diplomas	260 (40.1%)	257 (35.0%)	517 (37.4%)
Basic university degree	143 (22.1%)	162 (22.1%)	305 (22.1%)
Post graduate degree	129 (19.9%)	121 (16.5%)	250 (18.1%)
All adults	648(100%)	734(100%)	1382(100%)
**Family Income per month**:			
≤3000 SR	37 (6.4%)	41 (7.6%)	78 (6.9%)
>3000–5000 SR	82 (14.1%)	79(14.5%)	161 (14.3%)
>5000–10000 SR	173 (29.8%)	178 (32.8%)	351(31.2%)
>10000–20000 SR	172 (29.6%)	137(25.2%)	309(27.5%)
>20000 SR	117 (20.1%)	108 (19.9%)	225(20.0%)
All adults	581(100%)	543 (100%)	1124 (100%)
**Ethnicity**			
Arabian tribes	506 (79.6%)	546 (76.2%)	1052 (77.7%)
Sub-saharan African tribes	27 (4.2%)	35 (4.9%)	62 (4.6%)
Mediterranean Arab countries	35 (5.5%)	58 (8.1%)	93 (6.9%)
Indian subcontinent	39 (6.1%)	37 (5.2%)	76 (5.6%)
Central Asia	21 (3.3%)	24 (3.3%)	46 (3.4%)
South East Asia	8 (1.3%)	17 (2.4%)	25 (1.8%)
All adults	636 (100%)	717(100%)	1353(100%)
**Smoking status**:			
Non smoker	392 (59.9%)	629 (84.2%)	1022 (72.9%)
Former smoker	63 (9.6%)	21 (2.8%)	84 (6.0%)
Current smoker	199 (30.4%)	97 (13.0%)	296 (21.1%)
All adults	654 (100%)	747 (100%)	1401 (100%)
**Physical activity: (moderate intensity)**			
< 60 minutes / week	413 (62.2%)	536 (71.3%)	949 (67.0%)
60-<150 min/week	97 (14.6%)	64 (8.5%)	161 (11.4%)
≥150 mins/week	154 (23.2%)	152 (20.2%)	306 (21.7%)
All Adults	664 (100%)	752 (100%)	1416 (100%)

The majority of the participants were from Arabic tribes as expected. The age distribution reflects the growth in population of Saudi Arabia; adults aged < 30 years formed 43.1% of the participants, while only 8.1% were ≥60 years old.

The effect of age and sex on the prevalence of DM and prediabetes is presented in Table 2 Following blood testing to confirm diagnosis, 19 individuals previously unaware of their condition were found to be diabetic, and 122 were found to be prediabetic. The overall crude prevalence of DM and prediabetes was 15.7% and 10.2% respectively. The prevalence of DM was slightly lower among women than men, but the difference was not statistically significant (p = 0.58).

**Table 2:** The prevalence of diabetes mellitus (DM), and prediabetes according to age and sex.

	Men (Total N = 667) (% of age group)		Women (Total N = 752) (% of age group)	
	DM (N = 112)	Prediabetes (N = 69)	DM (N = 111)	Prediabetes (N = 76)
**Age group**:				
18–< 20	3.0	1.8	0.0	2.4
20–< 30	2.9	7.3	3.3	4.9
30–< 40	9.3	9.3	6.5	11.8
40–< 50	16.8	13.9	21.2	13.0
50–< 60	40.0	14.3	37.4	15.4
≥ 60	56.5	9.7	56.6	3.8
**Total %**	16.8%	10.3%	14.8%	10.1%

N: Number of persons

The prevalence of both DM and prediabetes increased with age; DM exponentially and prediabetes up to 60 years of age. Among people aged 18–39 years prediabetes was slightly more common than DM, while in people aged 40 years or older DM was much more common than prediabetes. Of people aged 50 years or over 46.1% of men and 44.4% of women had DM. Less than half of the people aged 50 years or over were normoglycaemic.

Following standardization for age and sex based on official national population survey (Central Department of Statistics and Information official site- Ministry of planning—population survey 2010- http://www.cdsi.gov.sa/yb45/Pages/Chapter2.htm—in Arabic), the overall prevalence for DM was 12.1% (95% Confidence Interval 10.7–13.5), 12.9% (10.7–13.5) in men and 11.4% (9.5–13.3) in women. The age and sex standardized prevalence of prediabetes was 9.0% (7.5–10.5), 9.4% (7.1–11.8) in men and 8.6% (6.6–10.6) in women. The prevalence based on "World Midyear Population by Age and Sex for 2012" as standard [20] (United States Census Bureau- International Programs) was 18.3% for DM and 11.9% for prediabetes.

Smoking was rare among women, but 30.4% of men were current smokers. The majority of the participants were engaged in less than one hour per week of intentional physical activity of moderate level, with just over the fifth of the population reaching the recommended duration of physical activity for at least 150 minutes/week.

All anthropometric, clinical, demographic and lifestyle variables were entered in stepwise logistic regression analysis model (Tables 3, 4 and 5). Demographic and lifestyle covariates were not associated significantly with DM either before or following adjustment (Table 5).

**Table 3:** Adjusted and unadjusted odds ratio (OR) for anthropometric and clinical covariates associated with prediabetes.

*Covariate*	Men				Women			
	Unadjusted OR (95%CI)	P	Adjusted OR (95%CI)	P	Unadjusted OR (95%CI)	P	Adjusted OR (95%CI)	P
**BMI categories**:								
Overweight (25-<30 kg/m^2^)^a^	0.86 (0.39–1.89)	0.711	0.49 (0.21–1.12)	0.092	2.48 (1.12–5.51)	**0.025**	2.02 (0.90–4.54)	0.091
Obese (≥30 kg/M^2^) ^a^	2.48 (1.27–4.86)	**0.008**	1.50 (0.73–3.08)	0.266	5.48 (2.65–11.33)	**<0.001**	3.74 (1.74–8.02)	**0.001**
**Abdominal obesity by WC**:								
Level 1 ^b^	3.65 (0.69–19.41)	0.129	2.35 (0.43–12.84)	0.323	6.99 (2.19–22.27)	**0.001**	5.64 (1.74–18.21)	**0.004**
Level2 ^b^	6.87 (1.64–28.75)	**0.008**	3.00 (0.68–13.3)	**0.049**	8.98 (3.19–25.30)	**<0.001**	6.05 (2.05–17.85)	**0.001**
**Risk by WC/HC**:								
**High Risk** (>0.95 for M, > 0.80 for F)^c^	3.00 (1.72–5.24)	**<0.001**	1.67 (0.89–3.10)	0.108	3.87 (1.87–7.99)	**<0.001**	2.93 (1.39–6.17)	**0.005**
**Risk by WC/Hgt**:								
**High Risk (WC:Hgt > 0.50)**^d^	5.62 (2.00–15.82)	**0.001**	3.04 (1.03–8.97)	**0.044**	8.67 (3.11–24.19)	**<0.001**	6.10 (2.12–17.61)	**0.001**
**% body fat classification according to age standards**:								
Overweight ^e^	1.83 (0.72–4.67)	0.205	1.36 (0.52–3.56)	0.529	1.64 (0.77–3.50)	0.201	1.25 (0.57–2.75)	0.573
Obese	2.68 (1.26–5.70)	**0.011**	1.63 (0.73–3.61)	0.232	2.38 (1.31–4.34)	**0.005**	1.52 (0.80–2.92)	0.205
**Diagnosed hypertension (140/90 or treatment)** ^f^	1.47 (0.71–3.07)	0.302	0.98 (0.35–2.74)	0.968	2.00 (1.01–3.96)	**0.047**	0.92 (0.25–3.33	0.894
**Diagnosed dyslipidemia**^g^	2.63 (1.35–5.12)	**0.004**	1.43 (0.71–2.92)	0.319	1.90 (0.96–3.76)	0.064	0.94 (0.43–2.03)	0.865
**Family history of diabetes (FHDM)**^i^	1.05 (0.60–1.85)	0.859	1.17 (0.65–2.10)	0.597	1.26 (0.74–2.14)	0.390	1.43 (0.83–2.47)	0.198
**Family history of dyslipidemia (FHDL)**^j^	1.13 (0.29–4.45)	0.864	0.95 (0.23–3.94)	0.938	1.77 (0.53–5.84)	0.352	1.97 (0.58–6.73)	0.281
**Family history of cardiovascular disease (FHCVD)**^k^	1.43 (0.75–2.73)	0.284	1.35 (0.69–2.63)	0.381	1.44 (0.81–2.55)	0.216	1.22 (0.68–2.21)	0.502

^a^ Reference is normal weight.

^b^ Reference is Level 1:W<94 cm for men, <80 cm F, Level 1:W<94 cm M, <80 cm for women.

^c^ Reference is low Risk (≤0.95 for men, ≤ 0.80 for women).

^d^ Reference is low Risk (WC:Hgt ≤ 0.50).

^e^ Reference is healthy % fat.

^f^ Reference is no hypertension.

^g^ Reference is no diagnosed dyslipidemia.

^i^ Reference is no FHDM.

^j^ Reference is no FHDL.

^k^ Reference is no FHCVD.

**Table 4:** Adjusted and unadjusted odds ratio (OR) for anthropometric and clinical covariates associated with type 2 diabetes.

	Men				Women			
	Unadjusted OR (95%CI)	P	Adjusted OR (95%CI)	P	Unadjusted OR (95%CI)	P	Adjusted OR (95%CI)	P
**BMI categories**:								
Overweight (25-<30 kg/M^2^) ^a^	2.35 (1.27–4.34)	**0.006**	1.12 (0.56–2.24)	0.757	5.53 (2.50–12.23)	**<0.001**	3.91 (1.57–9.70)	**0.003**
Obese (≥30 kg/M^2^) ^a^	3.64 (2.01–6.58)	**<0.001**	1.89 (0.97–3.70)	0.063	10.84 (5.09–23.06)	**<0.001**	5.36 (2.26–12.71)	**<0.001**
**Abdominal obesity by WC**:								
Level 1 ^b^	0.92 (0.15–5.67)	0.930	0.38 (0.06–2.52)	0.317	4.18 (1.23–14.21)	**0.022**	1.99 (0.57–7.02)	0.283
Level2 ^b^	8.38 (2.60–27.01)	**<0.001**	1.60 (0.46–5.62)	0.463	15.25 (5.52–42.12)	**<0.001**	3.45 (1.17–10.19)	**0.025**
**Risk by WC: HC**								
High Risk (>0.95 for M, > 0.80 for F)^c^	5.57 (3.54–8.76)	**<0.001**	2.23 (1.34–3.74)	**0.002**	4.49 (2.41–8.37)	**<0.001**	1.81 (0.92–3.58)	0.086
**Risk by WC: Hgt**								
High Risk (WC:Hgt > 0.50)^d^	7.86 (3.14–19.66)	**<0.001**	2.17 (0.81–5.84)	0.125	10.20 (4.09–25.41)	**<0.001**	2.58 (0.98–6.85)	0.056
**% body fat classification (Age adjusted)**:								
Overweight^e^	1.37 (0.63–2.99)	0.434	0.88 (0.37–2.11)	0.777	2.36 (1.20–4.66)	**0.013**	1.29 (0.60–2.78)	0.510
Obese^e^	2.98 (1.66–5.36)	**<0.001**	1.39 (0.71–2.72)	0.333	4.08 (2.36–7.06)	**<0.001**	1.56 (0.84–2.90)	0.157
**Diagnosed Hypertension (140/90 0r treatment)**^f^	6.31 (4.05–9.85)	**<0.001**	2.50 (1.50–4.17)	**<0.001**	11.89 (7.49–18.88)	**<0.001**	4.85 (2.87–8.20)	**<0.001**
**Diagnosed Dyslipidemia**^g^	9.77 (6.21–15.37)	**<0.001**	4.49 (2.73–7.37)	**<0.001**	8.18 (5.25–12.77)	**<0.001**	3.09 (1.86–5.14)	**<0.001**
**Diagnosed cardiovascular disease (CVD)**^h^	8.40 (3.67–19.23)	**<0.001**	2.66 (1.07–6.59)	**0.035**	5.00 (2.41–10.37)	**<0.001**	2.30 (0.96–5.53)	0.062
**Family history of diabetes (FHDM)**^i^	1.15 (0.76–1.74)	0.523	1.66 (1.02–2.70)	**0.043**	2.88 (1.77–4.70)	**<0.001**	3.86 (2.19–6.79)	**<0.001**
**Family history of dyslipidemia (FHDL)**^j^	2.23 (0.80–6.19)	0.123	2.22 (0.71–6.98)	0.173	13.2 (1.73–100.90)	**0.013**	18.18 (2.14–154.77)	**0.008**
**Family history of cardio-vascular disease (FHCVD)**^k^	1.27 (0.79–2.07)	0.326	1.20 (0.70–2.07)	0.515	1.60 (1.03–2.48)	**0.038**	1.33 (0.81–2.18)	0.263

^a^ Reference is normal weight.

^b^ Reference is Level 1:W<94 cm for men, <80 cm F, Level 1:W<94 cm M, <80 cm for women.

^c^ Reference is low Risk (≤0.95 for men, ≤ 0.80 for women).

^d^ Reference is low Risk (WC:Hgt ≤ 0.50).

^e^ Reference is healthy % fat.

^f^ Reference is no hypertension.

^g^ Reference is no diagnosed dyslipidemia.

^h^ Reference is no diagnosed CVD.

^i^ Reference is no FHDM.

^j^ Reference is no FHDL.

^k^ Reference is no FHCVD.

**Table 5:** Adjusted and unadjusted odds ratio (OR) for demographic and lifestyle covariates associated with prediabetes.

	Men				Women			
	Unadjusted OR (95%CI)	P	Adjusted OR (95%CI)	P	Unadjusted OR (95%CI)	P	Adjusted OR (95%CI)	P
**Educational Level**:								
≤ Primary school	Reference							
Intermediate school	0.08 (0.01–0.59)	**0.014**	0.17 (0.02–1.36)	0.094	0.56 (0.18–1.73)	0.313	0.50 (0.16–1.59)	0.240
Secondary school and diplomas	0.46 (0.22–0.96)	**0.039**	0.89 (0.40–1.98)	0.779	0.64 (0.27–1.51)	0.309	0.68 (0.28–1.61)	0.376
University degree	0.43 (0.18–1.01)	**0.048**	0.85 (0.34–2.11)	0.722	0.61 (0.24–1.54)	0.294	0.66 (0.26–1.71)	0.393
Post graduate degree	0.30 (0.11–0.84)	**0.021**	0.55 (0.19–1.60)	0.274	0.45 (0.16–1.24)	0.123	0.53 (0.19–1.48)	0.223
**Ethnicity**								
Arabian tribes	Reference							
Sub-saharan African tribes	0.70 (0.16–3.06)	0.637	0.78 (0.17–3.59)	0.752	0.39 (0.05–2.93)	0.358	0.29 (0.04–2.32)	0.244
Mediterranean Arab countries	1.31 (0.44–3.91)	0.630	1.25 (0.39–4.03)	0.706	0.58 (0.17–1.94)	0.375	0.54 (0.16–1.84)	0.322
Indian continent	0.82 (0.19–3.60)	0.790	1.28 (0.28–5.86)	0.747	0.66 (0.20–2.21)	0.498	0.51 (0.15–1.75)	0.286
Central Asia	1.40 (0.31–6.40)	0.663	1.73 (0.36–8.41)	0.495	0.95 (0.22–4.25)	0.951	1.00 (0.22–4.55)	0.994
South east Asia	2.45 (0.50–11.97)	0.267	1.67 (0.29–9.58)	0.563	0.00 (0.00–0.00)	0.999	0.00 (0.00–0.00)	0.999
**Smoking status**:								
Non smoker	Reference							
Former smoker	0.83 (0.41–1.67)	0.595	1.01 (0.49–2.09)	0.981	0.70 (0.35–1.43)	0.329	0.72 (0.35–1.47)	0.363
Smoker	1.51 (0.60–3.81)	0.385	1.66 (0.63–4.34)	0.305	1.16 (0.34–4.01)	0.816	1.24 (0.35–4.37)	0.743
**Physical activity**:								
< 60 minutes / week	Reference							
60-<150 min/week	0.76 (0.33–1.77)	0.522	0.77 (0.32–1.86)	0.565	1.08 (0.44–2.67)	0.863	1.05 (0.42–2.64)	0.911
≥150 mins/week	0.87 (0.44–1.73)	0.693	0.70 (0.34–1.43)	0.328	0.77 (0.39–1.52)	0.443	0.84 (0.42–1.69)	0.624

Following adjustments, prediabetes was significantly associated with general obesity in women, and with central obesity in both sexes. However, the association with WC was apparent already at the lower cutoff level for women, while in men it was significant only at the higher WC cutoff value. Furthermore, when the ratio of WC:HC was used to indicate abdominal obesity, a significant association was noted for women only, but the WC/height ratio remained significant in both sexes.

Similarly, DM was significantly associated with general obesity in women but not in men following adjustments. However, when WC was used to indicate central obesity, the association was significant only in women, but the ratio of WC:HC maintained significance in men, and the WC/height ratio lost statistical significance following adjustment. Furthermore, DM was also significantly associated with the presence of hypertension, dyslipidemia and FHDM in both sexes, as well as with the presence of CVD in men and with FHDL in women.

## Discussion

This study revealed a high prevalence of T2DM and prediabetes in the population of Jeddah, Saudi Arabia. This was expected based on the findings from previous studies [2, 21], however these were based on the measurement of FPG alone. Since FPG detects only part of people with DM and prediabetes [22–24], previous studies using FPG alone would be expected to seriously underestimate the magnitude of the problem of dysglycaemia in Saudi Arabia. Furthermore, most previous studies in Saudi Arabia have only included people with Saudi nationality, thus not giving a full picture of the population prevalence.

With diabetes mellitus reaching epidemic proportions in Saudi Arabia, it is imperative to initiate preventive programs to reduce the burden of T2DM in this population. Such programs should be based on adequate understanding of the magnitude of the problem, and the evaluation of prevention programmes also need to be based on proper epidemiological methods. Hence, we aimed at assessing the prevalence of DM and prediabetes in the population of the city of Jeddah, as well as identifying the factors associated with variation in prevalence. These data are important for the formulation of hypotheses of the causation of the DM in this population and for the development of appropriate intervention strategies to prevent DM, as suggested in the "European Evidence-Based Guideline for the Prevention of Type 2 Diabetes" [7], and the toolkit developed to implement these guidelines [8].

The city of Jeddah, with a population approaching 3.5 millions, is the most densely populated city in the kingdom. Being the gate way to the two holy cities in Islam, with many Muslims from different regions of the world settling there, its inhabitants form a good representative sample of the different ethnicities formulating the actual Saudi population. Therefore, by choosing Jeddah to perform our study, we hoped to ensure that most socioeconomic sectors and ethnicities living in Saudi Arabia are covered.

Some surveys were conducted previously [2, 21] on a national level. However; even though they provided useful preliminary information; they covered only some limited risk factors previously reported to be associated with DM; or example, family history of DM was missing. In addition, detailed data on habitual dietary intakes were not collected. Our survey attempted to fill this gap by including most known and also putative risk factors in the data collection. Furthermore, medical students; trained by community health consultants; performed data collection and carried out anthropometric measurements and that of BP, recording all medications and supplements used to ensure reliability of the data collected. Individuals suspected to have DM or prediabetes at screening were re-tested using standard methods (FPG and HbA1c) to determine the level of glycemia accurately. This ensured correct identification of DM and prediabetes, as well as other conditions. The use of WHO (EPI) cluster survey design ensured that the selected sample represents the population accurately. It is however not easy to verify our claim in view of the lack of published population data in Jeddah; but comparing our age and sex distribution with that published in The WHO NCD Risk Factors Standard Report, Saudi Arabia 2004 [21], no significant discrepancies were noted. All members of families aged ≥ 18 years living in the households selected were included in the study. This might explain the slightly lower number of men compared to women in contrast to what was found in the previous surveys [2, 21], since men living alone were not included in our sampling frame.

The young nature of the Saudi adult population is obvious from the results; more than 60% were less than 40 years of age, and mean age ± standard deviation of 35.7 ± 15.44 years, as reported in various earlier studies [21, 25]. Earlier studies on DM prevalence did not include younger adults as our study did, with the national study by Al-Nozha and his group [2] only included adults aged 30-70-years. Therefore, our calculated overall prevalence of DM following standardization for age in the adult population ≥ 18 years of age was somewhat lower than reported by other groups [2, 26]. However, the high prevalence in Saudi Arabia found by our study was demonstrated by the fact that over 40% of men and women aged 50 years or over had DM. Furthermore, earlier studies focused on DM only, while our study investigated the prevalence of prediabetes also. This allowed us to compare factors associated with each condition, and will aid in formulating preventive strategies to stop or at least delay the progression from prediabetes to DM.

Age was found to be the strongest predictor of diabetes and prediabetes in our study, as previously reported in other parts of the world [27–29]. Aging is known to be associated with increased adiposity and decreased muscle mass due to the usually noted decrease of physical activity. Such changes are reported to lead to a decrease insulin sensitivity [30, 31], predisposing individuals to metabolic syndrome or prediabetes [32–34]. Therefore, prevalence is expected to increase as the younger age groups (< 40 years) age in the coming years, if serious steps are not taken. Compared with the data recently published from the US using the same criteria as ours where the prevalence of DM was in the age group 45–64 years 16.2%, and in the group aged 64 years or over 24.7% [35], our results showed a much higher prevalence in Saudi people aged 50 year or over. Almost half of the people aged 50 years or over had DM in our study, and only less than half were classified as normoglycaemic. A recent Chinese study that used the same diagnostic criteria as our study found the prevalence of DM of 6.9% in people aged 18 or over and 12% in people aged 60 or over [36]. The Turkish national diabetes survey using the standard oral glucose tolerance test (FPG and 2-hour PG) reported in 2013 an overall age-standardized prevalence of 16.5% in people aged 20 years or above, and 25–40% prevalence in those aged 60 years or over [37]. While the Chinese had a lower prevalence of DM, the Turkish results are comparable to ours.

We found that obesity and, in particular, abdominal obesity is the second most important predictor of both DM and prediabetes. Obesity is well documented to be associated with increased risk of various chronic diseases, including T2DM [21, 30], and since it is a modifiable factor it should receive major attention in any future DM prevention programs [38]. A limitation in our study is that we used commonly used cutoff values for BMI to define overweight and obesity. These might not be the most suitable ones for the Saudi population, since the relationship between percentage body fat and BMI may be different among different ethnic groups [39]. Similarly, we used European cutoff values of abdominal obesity. Future study should also investigate local cutoff value for abdominal obesity, since cutoff values are dependent on ethnicity [40, 41], and using a non-ethnicity specific cutoff value might lead to misclassification [42].

Furthermore, associations with different indices of abdominal obesity were different between men and women. This has been noted in other epidemiological studies [43]. In our study, increased WC was strongly associated with prediabetes in both sexes, but only in women for DM. Similarly, WC: height ratio retained significant effect in both sexes in regression analysis in prediabetes, but not in DM. Therefore, the sex difference should be considered when assessing abdominal obesity as part of general risk assessment. Routine measurement of WC is unfortunately not carried out in most clinics. This must be rectified, and recommendations to include WC as part of routinely performed clinical assessments should be emphasized.

Family history of diabetes is another non- modifiable factor that remained strongly associated with DM (but not prediabetes) in our study following regression analysis in both sexes. Therefore, obese individuals with a family history of DM should receive special attention to prevent their progression to DM. It has been shown that prediabetic people with positive family history benefit from lifestyle intervention [44].

DM but not prediabetes was also significantly associated with the presence of hypertension, and dyslipidemia in both sexes. Whether hypertension and/or dyslipidemia preceded DM cannot be established from our study, which is another limitation inherent to all cross sectional studies. However, the lack of association between prediabetes and hypertension suggests that hypertension precedes the development of DM, and prediabetes people with hypertension will develop DM. Similar arguments can be proposed for dyslipidemia. Hypertension is highly prevalent among Saudi adults 30–70 years of age, affecting a quarter of the population [45]. Increased blood pressure is an important component of the metabolic syndrome reported to increase the risk of DM and cardiovascular disease [33, 46]. The prevalence of dyslipidemia is also high among Saudis [47], with hypertriglyceridemia affecting 44% of studied adult population. Lipotoxicity affects islet function adversely [48], and increased triglycerides are considered one of the components of metabolic syndrome increasing cardiometabolic risk of affected persons. Metabolic and hormonal changes in obesity lead to both increased blood pressure [49], and disturbance in lipid metabolism [50], hence it was not surprising to find the association between hypertension, dyslipidemia and DM. The effects of obesity need time to become apparent, so it was not noted in people with prediabetes. Obesity seems to be the main culprit in the development of both DM and prediabetes, and its long term effects might have mediated the association of DM with hypertension and dyslipidemia. There is a need to establish national cut off values for measures of obesity, and to reevaluate association with demographic and lifestyle factors once they are developed.

None of the demographic and lifestyle covariates retained association with DM or prediabetes following regression analysis. Sedentary lifestyle has been reported to induce insulin resistance in healthy volunteers [51], and has long been associated with increased risk of developing diabetes [52]. Therefore, finding no association of physical activity with prevalence of DM or prediabetes could be due to the design of the study. Single point estimations such as in our study might miss associations because they do not cover past practices. Similar explanations can be extended to smoking status. The lack of association of DM or prediabetes with ethnicity could be due to the mixed ethnicity of many participants. In Saudi Arabia, and especially in our region mixed ethnicities is very common, and participants in our survey wrote the father's ethnic origin as theirs, neglecting the mother's ethnicity. As is well established, genetic factors also appear to be important for the risk of DM, with family history of DM showing an association in both sexes.

One of the limitations of our study is that we did not use an oral glucose tolerance test, and thus cases of DM and prediabetes who have isolated high post-challenge glucose were not identified. In the US population aged 20 years or over, the unadjusted prevalence using the either HbA1c, FPG, or 2-hour PG definitions was 14.3% for total diabetes, and 38.0% for prediabetes in 2011–2012.- The unadjusted prevalence of total diabetes using the HbA1c or FPG definition only was 12.3%, and that of prediabetes 36.5% [52]. Thus, the true prevalence of DM and prediabetes in our study may be approximately 2% higher than we have found.

In conclusion, our study is the first one to study both the prevalence of prediabetes and DM in the Saudi population, and to investigate association with factors previously reported to be implicated in their etiology. We are also the first to report standardized prevalence estimates by age and sex for this population. While the prevalence of DM is not particularly common in young adults, the prevalence in people aged 50 years or older is very high, and it seems that the lifetime risk of DM is close to 50%. Prevention of T2DM should have a high priority in public health development in Saudi Arabia.
